# Protocol Biopsies in Kidney Transplant Recipients: Current Practice After Much Discussion

**DOI:** 10.3390/biomedicines13071660

**Published:** 2025-07-07

**Authors:** Christina Lazarou, Eleni Moysidou, Michalis Christodoulou, Stamatia Stai, Georgios Lioulios, Efstratios Kasimatis, Asimina Fylaktou, Maria Stangou

**Affiliations:** 1 School of Medicine, Aristotle University of Thessaloniki, 56429 Thessaloniki, Greece; lachristine91@yahoo.com (C.L.); moysidoueleni@yahoo.com (E.M.); michalischristodoulou22@gmail.com (M.C.); staimatina@yahoo.gr (S.S.); pter43@yahoo.gr (G.L.); frasci@outlook.com.gr (E.K.); 2 1st Department of Nephrology, Hippokration Hospital, 54642 Thessaloniki, Greece; 3 Department of Immunology, National Peripheral Histocompatibility Center, Hippokration Hospital, 54642 Thessaloniki, Greece; fylaktoumina@gmail.com

**Keywords:** kidney transplantation, protocol biopsy, immunosuppression, prognosis, acute rejection, chronic rejection

## Abstract

Protocol biopsies are a fundamental component in the management of kidney transplant recipients, offering critical insights into graft health by detecting subclinical pathological changes undetectable through routine clinical and laboratory assessments. Conducted at predetermined intervals, these biopsies enable early identification of subclinical rejection, chronic allograft nephropathy, drug-induced toxicities, viral infections such as BK polyomavirus nephropathy, and recurrence of primary glomerular diseases. Early detection facilitates timely therapeutic interventions, including immunosuppressive regimen adjustments, which are pivotal in preserving graft function and improving long-term outcomes. While the optimal timing and frequency of protocol biopsies vary, early post-transplant biopsies within the first year are widely advocated. High-risk groups, including ABO- and HLA-incompatible recipients and those with recurrent primary nephropathies, particularly benefit from surveillance biopsies. Despite the invasive nature and associated risks of biopsy procedures, most experts agree that the benefits outweigh the risks in selected populations. However, the role of routine protocol biopsies in low-risk patients remains debated due to unclear long-term outcome improvements and resource considerations. Retrospective observational studies have demonstrated the ability of protocol biopsies to detect subclinical pathological changes such as rejection, drug toxicity, viral infections, and recurrent diseases before clinical or laboratory abnormalities appear. These studies also highlight the impact of biopsy-guided interventions on graft survival and management in high-risk groups (e.g., HLA- and ABO-incompatible recipients, and patients at risk for disease recurrence). Furthermore, randomized controlled trials provide higher-level evidence showing that protocol biopsy-guided interventions improve graft function, reflected by better serum creatinine levels and glomerular filtration rates, compared to indicated biopsies alone. They also emphasize the importance of both early and late surveillance biopsies for predicting long-term outcomes. Expert opinion and consensus acknowledge the benefits of protocol biopsies for early detection and tailored management but also highlight ongoing debates regarding their routine use in low-risk patients due to risks, costs, and resource considerations. Overall, protocol biopsies represent a valuable tool for personalized graft monitoring and management, aiding in early detection of complications, guiding immunosuppressive therapy, and enhancing graft longevity. Further multicenter randomized trials are needed to refine guidelines and optimize their clinical utility.

## 1. Introduction

Protocol biopsies are fundamental in managing kidney transplant recipients, offering a proactive means to monitor graft histology and detect subclinical pathological changes that may jeopardize long-term outcomes [1,2,3,4,5,6]. These biopsies, performed at predetermined intervals regardless of clinical graft function, enable early identification of subclinical rejection, chronic allograft nephropathy (CAN), drug-induced toxicities, BK virus nephropathy, and recurrent glomerular diseases, which are conditions often undetectable by routine clinical or laboratory assessments [3,4,5,7,8]. Early detection facilitates timely interventions, including immunosuppressive therapy adjustments and antiviral treatments, which help preserve graft function [9].

Historically, protocol biopsy schedules extended up to 10 years post-transplant, with biopsies at implantation and regular intervals [6,8,10]. Contemporary practice emphasizes early post-transplant biopsies, particularly within the first year, to detect subclinical rejection and optimize immunosuppression [8,10]. Typical time points include zero-time or immediate post-reperfusion biopsies, three-month, and one-year biopsies, with some centers continuing surveillance at three, five, seven, or ten years to monitor chronic antibody-mediated rejection and late complications [9,10]. Frequency and timing are tailored to patient risk, immunosuppressive regimens, and institutional protocols [11].

The primary objective of protocol biopsies is detecting subclinical rejection (SCR)—histological rejection without clinical signs or renal function decline—which, if untreated, contributes to chronic graft injury and impaired survival [9,11,12]. High-risk patients, such as those with positive crossmatches, ABO incompatibility, high sensitization, or delayed graft function, benefit from more frequent biopsies [13,14]. Protocol biopsies improve long-term outcomes by enabling early treatment of SCR and other pathologies, thus preserving graft longevity [13,14,15].

This article presents the rationale for protocol biopsies in kidney transplant recipients, emphasizing their role in early detection of subclinical pathological changes and guiding timely therapeutic interventions to preserve graft function and improve long-term outcomes [1]. It aims to provide a current and comprehensive overview of the practice of protocol biopsies, focusing on updated timing and frequency, advantages and disadvantages, and particularly on high-risk groups such as ABO- and HLA-incompatible recipients and those at risk for recurrence of primary nephropathies. It also highlights the need for further multicenter randomized trials to refine guidelines and optimize clinical utility [1].

Despite their benefits, debate persists regarding routine protocol biopsy use solely for SCR detection due to procedural risks like bleeding and infection [13,15,16]. However, most experts agree that the benefits outweigh the risks, especially in high-risk groups [13,15].

Thus, the distinguishing aspect of this review appears to be its emphasis on recent evidence and tailored application in high-risk populations and synthesis of current controversies and recommendations for future research.

## 2. Role and Timing of Protocol Biopsies

Early biopsies post-transplant reveal a high incidence of SCR, with studies reporting over 40% of stable-function patients exhibiting subclinical tubulitis [12]. Such findings underscore the under-recognition of rejection in routine practice and justify systematic biopsy monitoring at intervals such as one week, one month, and one year [12]. Biopsies also assess recurrent glomerular diseases (e.g., IgA nephropathy) and nephrotoxicity from immunosuppressants like calcineurin inhibitors, allowing regimen modifications to mitigate injury [10]. Additionally, BK virus nephropathy can be detected early to prevent chronic damage [10,17].

Randomized controlled trials demonstrate that protocol biopsy-guided interventions improve graft function, reflected in lower serum creatinine and better glomerular filtration rates compared to indicated biopsies alone [18]. Furthermore, late protocol biopsies (12–24 months post-transplant) predict long-term graft outcomes more reliably than early biopsies (3–9 months), highlighting the need for both early and late surveillance [19].

Zero-time or reperfusion biopsies assess donor kidney quality, identifying acute injuries and chronic lesions predictive of graft survival [20,21]. Although some argue against routine use for donor discard decisions due to insufficient evidence, these biopsies provide valuable baseline data and inform post-transplant management [21].

In cases of delayed graft function (DGF), early biopsies within 7–10 days are critical to identify acute rejection or acute tubular necrosis, guiding management [22,23]. While some studies question the yield of routine biopsies in all DGF cases, biopsy decisions should be individualized based on clinical context and patient risk [23].

The incidence of SCR varies widely (2.6% to 25%) due to differences in immunosuppression and biopsy protocols. Although acute rejection rates have decreased and one-year graft survival is high (>95%), long-term graft survival improvements remain modest. SCR’s prognostic significance is crucial for guiding surveillance biopsy programs and treatment strategies to prolong graft longevity, despite challenges such as study heterogeneity and limited data [5].

Given below is a table(Table 1) that provides insights into the clinical recommendations for each biopsy time point.

## 3. Advantages and Disadvantages

Given below is an extensive list of the advantages and disadvantages of kidney allograft protocol biopsies:

### 3.1. Advantages of Kidney Allograft Protocol Biopsies

I. Early Detection of Subclinical Rejection
  - Protocol biopsies can identify subclinical acute rejection (SCAR) and other histological changes that may not present with overt clinical symptoms, allowing for timely intervention [2,6,7,11,12,27,28,29,30,31,32].
II. Diagnosis of Rejection:
  - Biopsies are considered the ‘gold standard’ for diagnosing acute rejection, crucial for establishing the cause of allograft dysfunction and allowing for accurate histopathological diagnosis [1].
III. Identification of Subclinical Pathologies:
  - In stable allografts, protocol biopsies can reveal unsuspected acute and chronic pathologies that may have significant prognostic implications for long-term graft outcomes [1,2].
IV. Predictive Value for Graft Function:
  - Findings from protocol biopsies, particularly at critical intervals such as six months post-transplant, correlate with long-term graft function, helping to identify patients at high risk for renal function deterioration [1,6,7,11,32].
V. Assessment of Immunosuppression Efficacy:
  - Biopsies can monitor the efficacy and toxicity of immunosuppressive therapies, guiding necessary adjustments to optimize treatment [1,2,11,28].
VI. Identification of Chronic Pathology:
  - Chronic transplant nephropathy and other chronic changes can be detected early through protocol biopsies, facilitating timely interventions that may prevent long-term graft dysfunction [1].
VII. Guidance for Therapeutic Adjustments:
  - Biopsy findings can guide clinicians in modifying immunosuppressive regimens, such as increasing dosages or changing medications, to better control immune responses [6,7,12,30].
VIII. Monitoring Graft Health:
  - Regular biopsies allow for ongoing assessments of graft histology, enabling the early identification of issues before they develop into significant problems [27,29,31].
IX. Standardization of Diagnosis:
  - Protocol biopsies contribute to the establishment of standardized histological criteria for diagnosing allograft rejection, which aids in consistent treatment approaches [27,28,32].
X. Research and Data Collection:
  - The data collected from protocol biopsies enhance understanding of the mechanisms of rejection and the effects of different immunosuppressive regimens, contributing to ongoing research in transplant medicine [29,30,32].
XI. Correlation with Long-Term Outcomes:
  - Histological findings from biopsies have been shown to correlate with long-term graft survival, thereby aiding in prognostic assessments [6,11].
XII. Improvement in Graft Function Over Time:
  - Regular monitoring through biopsies has been associated with improvements in graft histology and function [6,7,11,30].
XIII. Assessment of Anti-Donor Antibodies:
  - Protocol biopsies can help monitor the presence of donor-specific antibodies (DSA), indicating ongoing alloimmune processes [29].
XIV. Patient Stratification:
  - Biopsies can help stratify patients based on their risk of graft loss, allowing for tailored follow-up care and management strategies. The histologic findings from protocol biopsies provide prognostic information that is independent of graft function and other clinical parameters. This means they can identify patients at high risk for graft loss who may benefit from targeted therapeutic interventions. Conversely, protocol biopsies can also help identify patients at very low risk for graft loss, who might be candidates for modifications in their immunosuppressive regimens [6,12,31].
XV. Understanding Natural History
  - Protocol biopsies contribute to a better understanding of the natural history of transplant rejection, including the prevalence and progression of SCR over time. A study performed by Nankivell et al. reported a prevalence of SCR at various intervals post-transplant (e.g., 60.8% at 1 month, 45.7% at 3 months) [30].

### 3.2. Disadvantages of Kidney Allograft Protocol Biopsies

I. Invasiveness:
  - Biopsies are invasive procedures that carry inherent risks, including bleeding, infection, and potential damage to the allograft. Some non-major safety events that have been reported are transient hematuria, arteriovenous fistula, urinary tract infection and wound infection [1,6,7,9,27,29,30,31,32].
II. Risks Associated with Biopsy Procedures:
  - Complications such as hemorrhage and infection may occur, with reported serious complication rates around 0.4% [12,28].
III. Interpretation Challenges:
  - Variability in the interpretation of biopsy results can complicate the distinction between clinical and subclinical rejections, leading to potential mismanagement [1,2,7,27,29,31,32].
IV. False Sense of Security:
  - Reliance on serum creatinine levels for assessing allograft function may result in underappreciation of ongoing inflammation, as subclinical rejection can occur without significant changes in creatinine [1,11].
V. Sampling Error:
  - There is a possibility that the biopsy may not accurately represent the overall condition of the graft, leading to missed diagnoses [6].
VI. Potential for Overdiagnosis:
  - Minor histological changes may be overinterpreted as clinically significant, resulting in unnecessary interventions or overtreatment [6,27,29,30].
VII. Uncertainty Regarding Treatment Necessity:
  - There is ongoing debate about whether treating subclinical findings is necessary, as some experts argue that intervening in asymptomatic conditions may be unwarranted and potentially harmful [6,12,28].
VIII. Cost and Resource Allocation:
  - Regular protocol biopsies may impose significant healthcare costs and resource utilization, which could be a limitation in some settings [1,6,7,11,28,29,30].
IX. Psychological Impact on Patients:
  - The knowledge that biopsies are being performed regularly can induce anxiety in patients regarding their graft status and the possibility of rejection [7,28,30].
X. Limited Clinical Relevance:
  - Some histological findings may not translate into clinically significant outcomes, raising questions about the necessity of treatment for mild changes [30,32].
XI. Need for Standardization:
  - Existing classification systems for acute rejection, such as the Banff criteria, require further standardization and development to enhance diagnostic accuracy [1].
XII. Potential for Overtreatment:
  - The detection of subclinical rejection may lead to unnecessary increases in immunosuppressive therapy, exposing patients to higher risks of side effects, as well as malignancies and infections [12,30,32].
XIII. Variability in Histological Interpretation:
  - Differences in observer experience and the subjective nature of histological assessment can lead to inconsistent interpretations of biopsy results. There is potential for misinterpretation of biopsy results, leading to false positives or negatives and, therefore, misleading results. This can result in unnecessary anxiety for patients or inadequate management of actual rejection episodes [2,11,27,30,31,32].
XIV. Resource-Intensive:
  - The necessity for obtaining multiple biopsies during the initial year can be resource-demanding, requiring careful coordination among medical personnel and facilities, which consequently leads to substantial healthcare expenditures [11,28,30].
XV. Unclear Benefit:
  - The concrete benefits of routine protocol biopsies in improving long-term outcomes remain debatable, with some studies indicating no significant advantages [2,32].

This comprehensive list encapsulates the multifaceted considerations surrounding the use of protocol biopsies in kidney transplantation, highlighting both their potential benefits and inherent challenges.

Disadvantages based on categories (cost/resource burden interpretation variability, etc.) are as follows:

I. Procedural Risks and Invasiveness
  - Protocol biopsies are invasive procedures carrying inherent risks such as bleeding, infection, and potential damage to the allograft [1,6,7,9,27,29,30,31,32].
  - Non-major safety events reported include transient hematuria, arteriovenous fistula, urinary tract infection, and wound infection [12,28].
  - Serious complication rates are approximately 0.4%, including hemorrhage and infection [12,28].
II. Interpretive and Diagnostic Limitations
  - Variability in the interpretation of biopsy results can complicate distinguishing between clinical and subclinical rejection, potentially leading to mismanagement [1,2,7,27,29,31,32].
  - Differences in observer experience and the subjective nature of histological assessment may cause inconsistent or misleading results, resulting in false positives or negatives [2,11,27,30,31,32].
  - Minor histological changes might be overinterpreted as clinically significant, leading to unnecessary interventions or overtreatment [6,27,29,30].
  - Existing classification systems, such as the Banff criteria, require further refinement and standardization to improve diagnostic accuracy [6,27,29,30].
III. Resource and Cost-Related Limitations
  - Routine protocol biopsies impose significant healthcare costs and require considerable resource utilization, including coordination among medical personnel and facilities [11,28,30].
  - The necessity for multiple biopsies, especially in the initial post-transplant year, can be resource-demanding and financially burdensome [1,6,7,11,28,29,30].
  - Psychological impact on patients is also a consideration, as regular biopsies may induce anxiety regarding graft status and potential rejection [7,28,30].
  - The overall cost-effectiveness of routine protocol biopsies remains unclear, particularly in low-risk populations on modern immunosuppressive regimens [2,32].

Given below is Table 2 that presents the advantages and disadvantages of protocol biopsies at different time points.

## 4. The Role of Protocol Biopsies in High-Risk Kidney Transplant Recipients

Kidney transplantation has revolutionized the management of end-stage renal disease, offering improved quality of life and survival compared to dialysis. However, long-term graft survival remains a challenge, particularly in high-risk patient populations such as HLA-incompatible (HLAi) recipients, ABO-incompatible (ABO-I) recipients, and those with an increased risk of recurrence of primary nephropathy. Protocol biopsies, performed at predetermined intervals regardless of clinical indications, have emerged as a pivotal tool in the proactive monitoring of renal allografts. Below, we explore the role of protocol biopsies in these high-risk groups, underscoring their clinical utility, challenges, and impact on graft outcomes.

### 4.1. Protocol Biopsies in HLA-Incompatible Kidney Transplant Recipients

HLA incompatibility poses significant immunological challenges, increasing the risk of both acute and chronic rejection episodes. The sensitized immune status of HLAi recipients predisposes them to subclinical rejection (SCR) and antibody-mediated rejection (AMR), often undetectable through routine clinical and laboratory assessments. Surveillance protocol biopsies, therefore, are essential in this population to unveil early pathological changes and guide timely therapeutic interventions.

A landmark study evaluating protocol biopsies in a low-risk recipient cohort revealed critical findings, including subclinical rejection, chronic T cell- or antibody-mediated rejection, BK virus-associated nephropathy, and calcineurin inhibitor toxicity, with management changes implemented in 56% of cases based on biopsy results [33]. Such findings emphasize the biopsies’ role in optimizing transplant management through early detection and intervention [33].

Specifically in HLAi recipients, a longitudinal cohort study involving 129 patients undergoing desensitization for donor-specific antibodies (DSAs) demonstrated the importance of routine protocol biopsies at 1, 3, 6, and 12 months post-transplant [34]. These biopsies identified early microcirculation inflammation and subclinical rejection, which, if left untreated, could progress to transplant glomerulopathy (TxGN), a marker of chronic graft injury [34]. The early identification allowed for adjustments in immunosuppressive regimens, stabilizing renal function and enhancing graft survival [34].

Complementing these findings, a multicenter national cohort study comparing HLAi and HLA-compatible (HLAc) recipients underscored the critical role of protocol biopsies in early detection of AMR and other complications [35]. The study advocated for consistent monitoring using detailed records of DSAs and flow cytometry crossmatch (FC-XM) results to tailor patient care and mitigate risks inherent to sensitized populations [35].

In conclusion, protocol biopsies represent an indispensable component of post-transplant care in HLAi recipients [33,34,35]. Their capacity to detect subclinical immunological activity facilitates early, targeted interventions that improve long-term graft outcomes [33,34,35]. While cost–benefit analyses and challenges such as sampling errors remain areas for further research, the evidence strongly supports protocol biopsy integration in managing this high-risk group [33,34,35].

### 4.2. Protocol Biopsies in ABO-Incompatible Kidney Transplant Recipients

ABO incompatibility, once considered a contraindication to transplantation, has become more manageable with advances in desensitization protocols. Nevertheless, the immunological hurdles persist, with an increased incidence of subclinical rejection and antibody-mediated injury over time. Protocol biopsies, thus, serve a critical function in the surveillance and management of ABO-I transplant recipients.

Studies from centers such as the University Hospital Basel and Kyushu University Hospital highlight the importance of protocol biopsies in detecting subclinical rejection and guiding immunosuppressive therapy adjustments in ABO-I recipients [36,37,38,39,40]. A prospective analysis emphasized individual evaluation of biopsy results to refine monitoring strategies tailored to patient-specific risks [33]. Retrospective reviews reported comparable allograft pathology between ABO-I and ABO-compatible cohorts, but underscored the biopsies’ utility in identifying antibody-mediated rejection even with desensitization [35].

Regular protocol biopsies in ABO-I recipients are particularly warranted in patients with combined ABO and HLA incompatibilities, who represent a subset at heightened immunological risk [36,37,38,39,40]. By enabling early detection of graft inflammation and injury, these biopsies allow clinicians to pre-emptively modify immunosuppressive regimens and mitigate chronic allograft damage [36,37,38,39,40]. Despite the evolving landscape of immunosuppression, the role of protocol biopsies remains firmly justified in the care of ABO-I and HLA-i transplant recipients, balancing the benefits of early pathology detection against procedural risks [36,37,38,39,40].

A systematic review and meta-analysis assessed clinical outcomes after ABOi-rTx compared to ABO-compatible renal transplantation (ABOc-rTx) by analyzing 40 observational studies including over 65,000 patients. The findings indicate that ABOi-rTx is associated with significantly higher mortality and lower graft survival during the early post-transplant period (up to five years), with these differences diminishing beyond eight years. ABOi-rTx recipients also experienced more infectious and surgical complications. Rituximab-based desensitization protocols improved outcomes but did not fully eliminate the early increased risks. The study concludes that while ABOi-rTx is a valuable option when ABO-compatible donors are unavailable, paired kidney exchange programs may be preferable to avoid ABO incompatibility risks. Optimizing immunosuppressive regimens and increasing awareness of complications are recommended to improve outcomes [41].

Table 3 presents quantitative data on biopsy-related complication rates, subclinical rejection incidence, and long-term graft survival statistics

#### Psychological Issues Related to Repeated Biopsies

While there is limited data specifically on anxiety from repeated protocol biopsies, the overall evidence indicates that anxiety is present but often at low to moderate levels. Proper information, comfort, sedation, and mental health support can significantly reduce the burden. Systematic anxiety screening and empathy-based care should be part of transplant protocols [42,43,44,45].

### 4.3. Protocol Biopsies in Recipients at Increased Risk of Recurrence of Primary Nephropathy

Recurrence of primary kidney diseases such as focal segmental glomerulosclerosis (FSGS), IgA nephropathy (IgAN), membranous nephropathy (MN), and membranoproliferative glomerulonephritis (MPGN) is a significant cause of allograft loss. These diseases often recur silently before clinical manifestations emerge, underscoring the need for vigilant surveillance.

Retrospective observational studies demonstrate that protocol biopsies facilitate early detection of recurrent glomerular diseases, often before laboratory abnormalities or graft dysfunction become apparent [46]. This early identification enables timely and disease-specific therapeutic interventions, including immunosuppressive adjustments that can significantly improve graft survival [46].

In primary hyperoxaluria (PH), protocol biopsies detect recurrent calcium oxalate crystal deposition, which occurs in nearly half of recipients despite a lack of clinical symptoms [47]. Early biopsy findings guide aggressive metabolic management, including plasma oxalate reduction via dialysis, immunosuppressive regimen optimization to avoid nephrotoxicity, and promotion of high urine output to prevent crystal aggregation, collectively preserving graft function [47].

For primary glomerular diseases such as IgAN, MN, FSGS, and MPGN, protocol biopsies reveal recurrence rates substantially higher than those detected by clinical monitoring alone, with histological recurrence rates reaching up to 60% in IgAN and 80% in MPGN type II [48,49]. In FSGS, where rapid recurrence can occur within weeks post-transplant, biopsies are critical for early intervention with therapies such as plasmapheresis, targeting the presumed circulating permeability factor [50]. Moreover, protocol biopsies assist in differentiating recurrent disease from rejection or other causes of graft dysfunction, guiding precise management [49].

Additionally, in patients with monoclonal gammopathy of renal significance (MGRS), protocol biopsies detect subclinical histologic recurrence, facilitating early intervention that may prolong graft survival [51].

Overall, protocol biopsies in recipients at risk of primary disease recurrence provide invaluable insights into graft pathology, enabling pre-emptive and tailored management strategies that significantly impact long-term outcomes.

Below, in Table 4, biopsy outcomes are categorized according to high-risk and low-risk patients 

#### 4.3.1. Types of Rejection and Banff Classification

According to the Banff criteria [52,53,54,55], types of rejection include active AMR, chronic active AMR, chronic inactive AMR, probable AMR, borderline acute TCMR, acute TCMR (IA, IB, IIA, IIb), and chronic TCMR (IA, IB, II). Furthermore, acute antibody-mediated rejection (AMR) can occur both early (<3 months) and late (>3 months) after transplant [56]. In addition, AAMR is subclassified into three types according to the type of tissue injury: type I, acute tubular necrosis (ATN)-like; type II, glomerular type, resembling thrombotic microangiopathy; and type III, vascular type with arterial inflammation [57].

Hyperacute rejection is a severe and immediate immune response that occurs within minutes to hours after transplantation [58]. It is characterized by widespread thrombosis of graft vessels due to pre-existing antibodies in the recipient’s blood targeting the donor organ [58]. It is triggered by the binding of high titers of anti-HLA antibodies to HLA type I molecules on the surface of the allograft’s endothelial cells, leading to direct tissue damage and the activation of the classical complement pathway, often accompanied by the immediate cyanosis of the graft, thrombosis of the blood vessels, and extensive tissue necrosis [59]. This process results in severe endothelial damage in the allogeneic transplant [59]. Specifically, the progressive release of heparan sulfate from the surface, mediated by the enzymatic cleavage of the protein core and glycosaminoglycan chains, leads to the loss of the endothelial barrier, which, in turn, results in thrombotic microangiopathy due to cell damage and consequent platelet aggregation and adhesion [60,61]. This type of rejection is rare today due to pre-transplant crossmatching and screening for donor-specific antibodies [59].

Delayed hyperacute or accelerated rejection (DHAR) is observed when there is an abrupt decline in urine output and graft tenderness occurring 3 to 14 days after transplantation [62]. This type of rejection is also associated with the presence of donor-specific antibodies, similar to hyperacute rejection, but manifests later in the post-transplant period [60]. It indicates an ongoing immune response against the graft, necessitating prompt evaluation and intervention. It is a severe type of acute humoral rejection that occurs within two weeks after ABO blood type-incompatible kidney transplantation [63]. Additionally, subclinical AMR is defined as immunohistological evidence of AMR in kidney transplant recipients with normal renal allograft function [61]. The term “acute vascular rejection” (AVR) is often ambiguously applied to all vascular lesions found during acute rejection. According to the Banff 2009 classification, AVR may fall into one of four categories: acute T cell-mediated rejection (ATMR) type IIA, ATM type IIB, ATMR type III, and acute antibody-mediated rejection (AAMR) type III [52,53,54].

#### 4.3.2. Staining Techniques

Staining techniques are fundamental to the pathological evaluation of renal allografts, providing critical information for diagnosis, disease classification, and management. Conventional histochemical stains such as Hematoxylin and Eosin (H&E) and Periodic Acid-Schiff (PAS) play essential roles in highlighting specific tissue components and cellular structures. H&E staining differentiates nuclei and cytoplasm, providing an overall view of tissue architecture, while PAS accentuates basement membranes, which is valuable for assessing glomerular and tubular structures in renal biopsies. Other important stains include Jones silver stain for basement membranes and Sirius Red for collagen in connective tissue, which aid in identifying fibrosis and chronic damage, thus being crucial for evaluating allograft rejection and progression of disease [61,64,65].

Immunohistochemical (IHC) staining techniques further enrich diagnostic precision by identifying specific cell types or molecular markers. In renal allografts, C4d staining—performed either by immunofluorescence (IF) or immunoperoxidase (IP)—is particularly important as a marker of antibody-mediated rejection (AMR). C4d is a complement split product that binds to endothelial surfaces in peritubular capillaries (PTCs), indicating the presence of donor-specific antibodies and classical complement pathway activation. Diffuse C4d positivity is highly sensitive (95%) and specific (96%) for donor-specific antibodies, making it a central diagnostic criterion for AMR when combined with serological data and histological findings. Notably, recent studies suggest that diffuse C4d positivity in early acute rejection does not necessarily predict worse clinical outcomes or steroid resistance when standardized treatment protocols are applied, challenging previous assumptions that C4d positivity alone mandates more aggressive immunosuppression [61,66].

Immunofluorescence remains the gold standard for detecting immunoglobulins and complement components in glomerular and extraglomerular regions, essential for diagnosing immune complex-mediated diseases and transplant pathology. Conventional IF on unfixed frozen sections offers high sensitivity and precise localization with minimal background. However, when fresh tissue is unavailable or inadequate, immunofluorescence on formalin-fixed, paraffin-embedded tissue (IF-P) with antigen retrieval serves as a valuable salvage technique. IF-P can reveal “masked” immune deposits missed by routine IF, aiding diagnosis in complex or atypical cases such as membranous-like glomerulopathy with masked IgG kappa deposits or monoclonal gammopathy-associated diseases. While IF-P has limitations, including weaker staining and reduced sensitivity for certain components like C3, its integration with IF-F, light microscopy, and electron microscopy ensures a more comprehensive and accurate assessment of renal allograft biopsies [66,67,68].

The combined use of staining methods—conventional histochemical stains, IHC including C4d, and immunofluorescence techniques—alongside electron microscopy provides a synergistic approach to diagnosing and understanding renal allograft pathology. This integrated strategy enhances the visualization of structural and immunological abnormalities, facilitating precise diagnosis, prognosis, and guiding therapeutic decisions in transplant nephrology [64,65,66,69].

In summary, the pathological evaluation of renal allografts relies heavily on a spectrum of staining techniques: conventional stains like H&E and PAS for morphological assessment; immunohistochemical methods including C4d staining for detecting AMR; and immunofluorescence techniques for identifying immune deposits. Together, these approaches form a comprehensive diagnostic toolkit essential for managing renal transplant patients effectively.

Differences in biopsy yield and utility among special populations (ABOi, HLAi, patients with increased risk for primary disease recurrence):

Regarding differences in biopsy yield and utility among these groups, the article highlights the following:

1. HLA-Incompatible Recipients:
  - These recipients face significant immunological challenges, with a predisposition to subclinical rejection (SCR) and antibody-mediated rejection (AMR) that often go undetected clinically.
  - Protocol biopsies at 1, 3, 6, and 12 months post-transplant have a high yield in detecting early microcirculation inflammation and SCR, which, if untreated, may progress to chronic damage such as transplant glomerulopathy.
  - Early biopsy detection facilitates immunosuppressive regimen adjustments, stabilizing renal function and improving graft survival.
  - Studies show management changes based on biopsy findings in over half of cases, reflecting high utility in guiding therapy [6,30,31,32].
2. ABO-Incompatible Recipients:
  - Despite advances in desensitization, there remains an increased incidence of subclinical rejection and antibody-mediated injury in ABO-I transplants.
  - Protocol biopsies detect these subclinical changes effectively and assist in tailoring immunosuppression.
  - Their yield in detecting antibody-mediated rejection despite desensitization is comparable to HLAi recipients.
  - Particularly high-risk are patients with combined ABO and HLA incompatibilities, where regular protocol biopsies are strongly indicated to prevent chronic damage by enabling early intervention [6,33,34,35,36,37].
3. Recipients at Risk of Primary Disease Recurrence:
  - Recurrence of diseases such as IgA nephropathy, FSGS, membranous nephropathy, and MPGN is a major cause of graft loss and often occurs silently.
  - Protocol biopsies reveal recurrence rates much higher than clinical monitoring alone (e.g., up to 60% in IgA nephropathy), enabling earlier and disease-specific interventions such as plasmapheresis in FSGS.
  - In primary hyperoxaluria, biopsies detect calcium oxalate deposition early, guiding metabolic management to preserve graft function.
  - This group benefits from high biopsy yield in detecting subclinical recurrence that directly impacts tailored therapeutic strategies and outcomes [38,39,40,46,47,48].
4. Impact on Therapeutic Outcomes:
  - Across these groups, early detection via protocol biopsy leads to timely therapeutic modifications (e.g., immunosuppression intensification, treatment of AMR, metabolic management), which have been shown to stabilize or improve graft function and enhance long-term survival [30,31,32,38,39].
  - For example, in HLAi recipients, early identification of microcirculation inflammation allowed for immunosuppressive adjustments that improved graft survival [31].
  - In ABO-I recipients, biopsies guide immunosuppressive therapy adjustments to mitigate antibody-mediated injury [33,34].
  - For disease recurrence, early diagnosis enables initiation of disease-specific therapies preventing further graft injury [38,47].

#### 4.3.3. The Emerging Role of Non-Invasive Biomarkers as Alternatives or Complements to Protocol Biopsies

There is a growing role of non-invasive biomarkers in detecting and monitoring kidney allograft dysfunction, based on their potential to supplement or, in some cases, reduce reliance on protocol biopsies. Biomarkers such as donor-derived cell-free DNA (dd-cfDNA), gene expression profiles (GEPs), and urinary chemokines (e.g., *CXCL9*, *CXCL10*) offer significant advantages, including earlier detection of graft injury compared to conventional markers like serum creatinine, reduced procedural risks, and the ability to monitor immune activity and treatment response over time [63,70,71,72,73,74,75,76]. Advanced tests like AlloMap, TruGraf, and composite panels (e.g., the “Q-score”) demonstrate high negative predictive value and the potential to minimize unnecessary biopsies by identifying early immune activation and subclinical rejection [71,72,77,78].

However, the use of non-invasive biomarkers is not without limitations. While sensitive, dd-cfDNA and urinary chemokines may lack specificity, as levels can rise due to infections or unrelated inflammatory conditions [73,79]. Technical challenges such as rapid degradation of urinary markers, lack of standardization, and the high cost and limited accessibility of advanced assays further hinder widespread adoption, particularly in resource-constrained settings [77,78,80]. Moreover, although these biomarkers enhance monitoring and risk stratification, they cannot fully replace biopsies, which remain essential for definitive diagnosis and classification of rejection severity [60,73]. Thus, biomarkers are best positioned as complementary tools within a broader diagnostic strategy.

## 5. Assessment of Chronic Allograft Nephropathy

Protocol biopsies are pivotal in early detection and monitoring of chronic allograft nephropathy (CAN), now termed interstitial fibrosis and tubular atrophy (IF/TA) [24,81]. They reveal subclinical rejection, inflammation, and tubulitis predictive of CAN and graft loss [24,82]. Longitudinal biopsy data characterize CAN progression and guide therapeutic adjustments to minimize calcineurin inhibitor toxicity and immunologic injury [24,25,26,82,83,84].

## 6. Post-Acute Rejection Monitoring

Protocol biopsies following episodes of acute rejection (AR) play a critical role in the diagnosis, management, and prognostication of kidney transplant outcomes. Their value lies in detecting subclinical rejection (SCR), which is histologically evident acute rejection without concurrent functional deterioration [85], typically precedes clinical rejection episodes, and is associated with subsequent chronic allograft damage [86]. Furthermore, persistent inflammation and SCR detected on protocol biopsies after treatment of acute rejection have prognostic implications for allograft survival [85,86].

Their role also encompasses evaluating the effectiveness of treatment following an episode of acute rejection [86]. By documenting whether histological signs of rejection have resolved or persist, these biopsies provide critical information that can inform decisions about the need for additional or adjusted therapeutic interventions [86]. Studies have shown that despite treatment (e.g., corticosteroids), subclinical rejection may persist in a significant proportion of patients, necessitating augmentation of immunosuppression [87]. In addition, follow-up biopsies can detect persistent SCR, borderline changes, or chronic histologic damage that may not be reflected by serum creatinine or other non-invasive markers [85].

Furthermore, the histologic chronicity score correlates better with long-term graft survival, than serum creatinine or eGFR [86]. They also help identify other causes of graft dysfunction such as calcineurin inhibitor nephrotoxicity or BK virus nephropathy [85,87].

Benefits are greatest in high-risk patients (e.g., those with preformed donor-specific antibodies or on steroid-sparing regimens) where subclinical rejection is more prevalent [86]. In low-risk patients on potent immunosuppression (e.g., tacrolimus-based triple therapy), the cost-effectiveness of routine protocol biopsies is less clear due to lower incidence of SCR [85,86].

Some centers perform protocol biopsies universally after transplant (including after rejection episodes) while others limit biopsies to high-risk groups or those with clinical indications [85]. Protocol biopsies guide escalation or modification of immunosuppressive therapy after acute rejection [87,88]. Follow-up biopsies are used to monitor treatment response and to detect persistent or recurrent subclinical rejection that could benefit from further intervention [85,87].

Overall, post-acute rejection protocol biopsies guide escalation or modification of immunosuppressive therapy after acute rejection, monitor treatment response, and detect persistent or recurrent subclinical rejection that may benefit from further intervention. They have a low complication rate and can prevent progression to chronic rejection and graft loss. While universal protocol biopsies may not be cost-effective in all populations, especially low-risk patients on modern immunosuppressive regimens, they are particularly important in high-risk recipients or those with atypical clinical courses. Thus, protocol biopsies should be performed following acute rejection episodes to ensure optimal management and long-term allograft survival, especially in centers with adequate resources and expertise [6,7,8,9,10,25,26,87,88].

**Figure 1:**
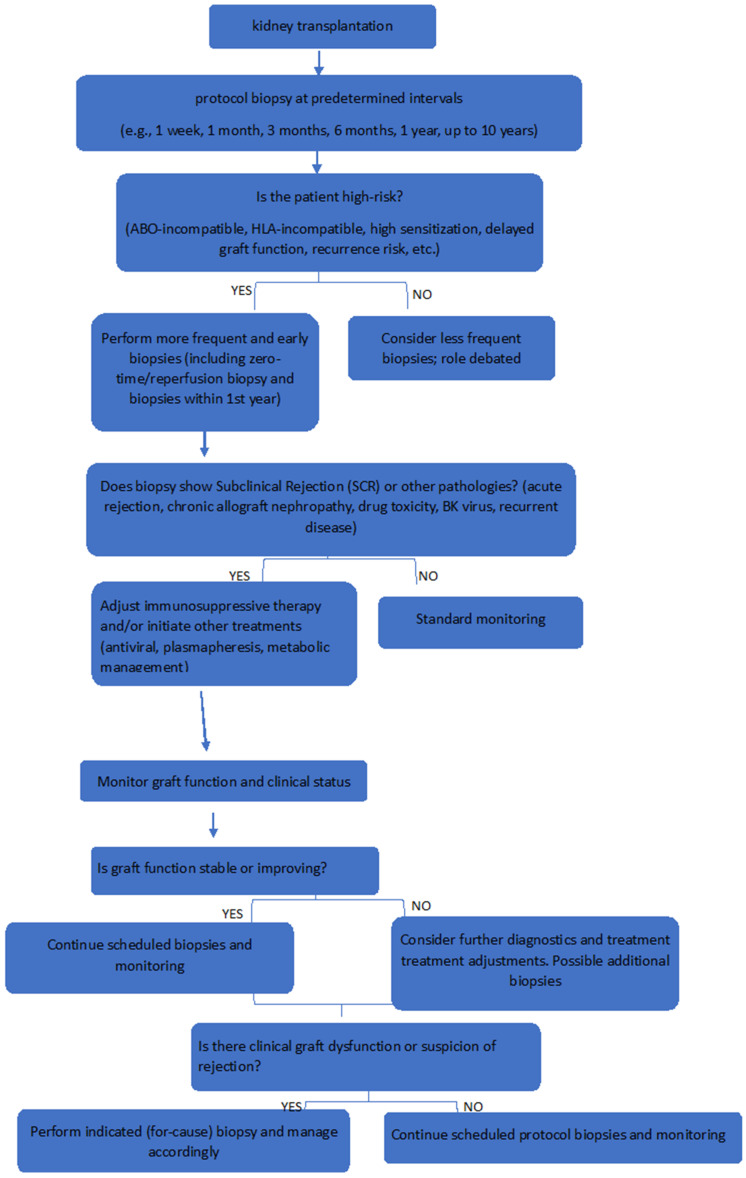
A decision-making flowchart summarizing biopsy indications by risk group.

A decision-making flowchart summarizing biopsy indications by risk group is shown in Figure 1.

### Risk–Benefit Ratio in Low-Risk Populations

Recent evidence evaluating the risk–benefit ratio of protocol biopsies in low-risk kidney transplant recipients indicates minimal clinical benefit alongside a non-negligible risk profile [9,13,89,90,91]. A 2024 systematic review and meta-analysis found no significant advantage of protocol biopsies over standard care in detecting or reducing acute rejection, preventing graft loss at 12 months, or improving GFR at 6 months, while biopsy arms experienced increased safety events [9]. A large single-center retrospective study similarly showed fewer rejection episodes identified by protocol biopsies without corresponding improvements in one-year graft survival or GFR, and noted small but real complication risks [89]. Guidelines, including the National Kidney Foundation, suggest protocol biopsies have limited utility in low-risk patients and are more appropriate for high-risk groups, emphasizing the logistic burden and patient discomfort of frequent biopsies without clear long-term benefits [13]. Safety data from a large cohort study demonstrate minor complication rates around 6% and major complications near 1%, confirming overall safety when performed correctly but not indicating that they are risk-free [90]. Contemporary interpretation supports a risk-adaptive approach—reserving biopsies for patients with heightened immunologic risk—and highlights emerging non-invasive biomarkers that could supplant universal biopsy strategies [91]. Thus, routine protocol biopsies in low-risk populations yield + minimal clinical advantage and carry modest procedural risks, favoring selective rather than universal application [9,13,89,90,91].

## 7. Conclusions

Protocol biopsies represent a critical component of kidney transplant management, enabling early detection of subclinical rejection, drug toxicity, antibody-mediated injury, viral infections, and recurrent diseases, thereby facilitating timely interventions to preserve graft function and improve long-term outcomes [1,2,3,4,5,6,9,12,13,14,15,18,33,34,35,46,47,48,85,86].

While procedural risks, interpretive variability, and resource implications, as well as costs, exist, in carefully selected high-risk populations, the benefits overwhelmingly justify their use since studies have concluded that their benefit is particularly evident in high-risk populations such as ABO- and HLA-incompatible recipients and those at risk for disease recurrence [13,15,33,34,35,36,37,46,48].

Selective application based on patient risk factors and evolving immunosuppressive protocols is recommended until further randomized controlled trials clarify their broad utility [9,10,18]. Future research, including multicenter randomized trials, is needed to refine biopsy protocols, minimize risks, and integrate emerging non-invasive diagnostic modalities.

Protocol biopsies are a vital element in the management of kidney transplant recipients, enabling the early detection of subclinical rejection, drug toxicity, antibody-mediated injury, viral infections, and recurrent diseases. These early identifications facilitate timely interventions that help preserve graft function and improve long-term outcomes. Although there are inherent risks, interpretative variability, and resource considerations involved, the benefits of protocol biopsies are particularly evident in carefully selected high-risk populations such as ABO- and HLA-incompatible recipients and those at risk for disease recurrence. Selective application based on patient risk factors and evolving immunosuppressive protocols is advisable until further randomized controlled trials clarify their broader utility. Moreover, future research should focus on cost-effectiveness studies and the integration of protocol biopsies with emerging molecular diagnostic tools to refine biopsy protocols, minimize risks, and enhance clinical decision-making in kidney transplantation [1,2,3,6,9,13,15,25,26,30,31,32,38,40].

## Figures and Tables

**Table 1:** Clinical Recommendations at each biopsy time point.

Biopsy Time Point	Clinical Recommendations
Zero-time/Immediate post-reperfusion	- Assess donor kidney quality, acute injuries, and chronic lesions predictive of graft survival [2,20,21]. - Provides baseline data to inform post-transplant management [2,20,21].
1 week post-transplant	- Early detection of subclinical rejection (SCR), especially subclinical tubulitis [2,12]. - Justifies systematic biopsy monitoring due to high incidence of SCR [2,12].
1 month post-transplant	- Continued surveillance for SCR and early pathological changes [2,10,12]. - Early intervention for subclinical rejection and nephrotoxicity from immunosuppressants [2,10,12].
3 months post-transplant	- Detect subclinical rejection, recurrent glomerular diseases, and drug toxicity [2,10,18]. - Optimize immunosuppressive regimens [2,10,18].
6 months post-transplant	- Predictive value for long-term graft function. - Identify patients at high risk for renal function deterioration [3,6].
12 months (1 year) post-transplant	- Detection of chronic antibody-mediated rejection and late complications [2,19,24]. - Assess chronic allograft nephropathy (CAN)/interstitial fibrosis and tubular atrophy (IF/TA) [2,19,24].
Beyond 1 year (3, 5, 7, 10 years)	- Continued surveillance for chronic rejection and late complications, especially in high-risk patients [2,10]. - Tailor frequency based on patient risk and institutional protocols [2,10].
Early biopsy within 7–10 days (in cases of delayed graft function—DGF)	- Identify acute rejection or acute tubular necrosis [2,22,23]. - Guide management in DGF cases, individualized based on clinical context and patient risk [2,22,23]
Post-acute rejection episodes	- Detect residual or persistent subclinical rejection [6,25,26]. - Assess treatment efficacy and early chronic allograft injury [6,25,26]. - Guide immunosuppressive therapy adjustments to prevent chronic rejection [6,25,26].

**Table 2:** Advantages and disadvantages of protocol biopsies at different time points.

Time Point	Advantages	Disadvantages
Zero-time/Reperfusion	Baseline assessment of donor kidney quality; detects acute injury; informs management	Invasive; limited evidence for donor discard decisions
Early (1 week to 6 months)	Early detection of SCR, drug toxicity, BK virus; guides timely therapy; predicts graft function	Procedural risks; sampling error; psychological impact; resource-intensive
Late (1 year and beyond)	Monitors chronic injury; predicts long-term outcomes; detects late rejection	Uncertain clinical relevance of mild findings; possible overtreatment; cost and resource use
Post-Acute Rejection	Detects residual SCR; guides therapy adjustments; prognostic value	Interpretation challenges; may not be cost-effective universally

**Table 3:** Quantitative data on biopsy-related complication rates.

Parameter	Data/Findings
Subclinical Rejection (SCR) Incidence	- Over 40% of stable-function patients exhibit subclinical tubulitis early post-transplant [2]; - Prevalence of SCR at various intervals: 60.8% at 1 month, 45.7% at 3 months post-transplant [3,4].
Biopsy-Related Complication Rates	- Serious complication rates around 0.4% [4]; - Reported non-major safety events: transient hematuria, arteriovenous fistula, urinary tract infection, wound infection [4].
Long-Term Graft Survival and Biopsy Findings	- Histological findings from protocol biopsies correlate with long-term graft survival [3]; - Late protocol biopsies (12–24 months post-transplant) better predict long-term outcomes than early biopsies (3–9 months) [2]; - Persistent subclinical rejection after treatment is associated with worse graft survival [8].
Impact of Protocol Biopsies on Management	- Management changes based on biopsy results implemented in 56% of cases in one low-risk cohort study [6]; - Early identification of subclinical rejection allows immunosuppressive regimen adjustments improving graft survival [6,7,8].

**Table 4:** Findings from protocol and for-cause biopsy on high and low-risk patients.

Risk Group/Patient Type	Biopsy Type Outcomes/Findings	Comments/Notes
High-Risk Patients (e.g., positive crossmatch, ABOi, HLAi, high sensitization, delayed graft function)	Protocol Biopsy - Early detection of subclinical rejection (SCR) and antibody-mediated rejection (AMR)	- Enables early immunosuppressive adjustments improving graft survival [1,2,4,13,15,30,31,32,33,34]
	For-Cause Biopsy - Detection only after clinical dysfunction or graft impairment	- Delayed detection; poorer long-term graft outcomes [1,13]
Low-Risk Patients	Protocol Biopsy - Detection of subclinical pathologies less frequent	- Cost-effectiveness and benefit less clear; routine use debated
	For-Cause Biopsy - Biopsy performed upon clinical indication	- May miss early subclinical rejection or pathology
